# Health Shock Effects on Diet: More Severe Shock—Stronger Response?

**DOI:** 10.1002/hec.4940

**Published:** 2025-01-22

**Authors:** Anna Kristina Edenbrandt, Kim Wadt Skak‐Hansen, Sinne Smed

**Affiliations:** ^1^ Department of Economics Swedish University of Agricultural Sciences Uppsala Sweden; ^2^ Department of Cardiology Rigshospitalet Copenhagen Denmark; ^3^ Department of Food and Resource Economics University of Copenhagen Copenhagen Denmark

## Abstract

We investigate whether the severity of lifestyle‐related health shocks affects the response in dietary patterns. Using data from official patient registers in Denmark, we analyze the effects from strong health shock (SHS) occurrences (cardiovascular disease) and mild health shock (MHS) occurrences (arterial hypertension and hypercholesterolemia). These data are combined with scanner data on food purchases obtained from a consumer panel. Our analysis examines dietary effects stemming from these health shocks, including various nutrients, food groups, and overall adherence to dietary guidelines. Our findings reveal immediate dietary responses to both severe and mild health shocks, with a larger effect observed for SHS compared to MHS. However, among individuals previously exposed to mild health shocks, we observe minimal to no alteration in food consumption after experiencing a SHS. We argue that failing to account for this potential self‐selection may lead to a misconception that severe health shocks do not result in dietary improvements.

## 1 Introduction

An unhealthy diet is a key risk factor for non‐communicable diseases such as diabetes, stroke and heart diseases (World Health Organization 2024), with cardiovascular diseases (CVDs) being the leading cause of deaths globally, accounting for one third of all deaths (World Heart Federation 2023). Arterial hypertension (measured by blood pressure) and hypercholesterolemia (measured by cholesterol levels), both which can be the consequence of an unhealthy lifestyle, increase the risk of CVDs. However, these risks can be reduced through healthy lifestyle choices, including a healthy diet low in salt and regular physical activity (World Health Organization 2023). Consequently, individuals diagnosed with arterial hypertension (HA) or hypercholesterolemia (HC) can reduce their risk of CVD by adopting these lifestyle modifications. A diagnosis with these conditions can hence be a signal about the potential consequences of unhealthy eating. Furthermore, individuals that suffer from CVDs can substantially decrease the risk of recurrence by adopting healthier lifestyles. The diagnosis of a CVD provides an even stronger signal about the consequences of unhealthy eating, which may influence the individual's dietary choices. As such, CVD, HA and HC serve as negative health shocks to the individual. Dietary recommendations are communicated through general information materials but are emphasized and directed toward the individual in conjunction with diagnosis or treatment.

A key question is if lifestyle‐related health shocks, along with the information provided in relation to these, are sufficient to bring about changes in food consumption in line with the recommended dietary patterns. We categorize HA and HC as comparatively milder negative health shocks. While these conditions are significant, they also serve as important causes of CVD, thereby functioning as warning signals for CVD onset. Intuitively, one might expect more severe negative health shocks to lead to more pronounced improvements in healthy eating habits, but this prediction ignores any potential self‐selection bias. We anticipate that some individuals suffering from lifestyle related CVD have experienced prior milder negative health shocks such as HA or HC yet failed to make (sufficient) changes to their lifestyles in response. Consequently, individuals suffering from CVD may be disproportionately represented by those who neglected to change behavior in relation to earlier mild negative health shocks, or warnings, such as diagnoses with HA or HC. This self‐selection bias challenges the assumption that behavioral changes are greater following a severe health shock compared to a mild one.

There is some evidence that individuals diagnosed with hypertension reduce their fat intake (Dai et al. 2022; Zhao, Konishi and Glewwe 2013). Carrera, Hasan, and Prina (2020) find that cholesterol test results influence employees spending in the workplace cafeteria, but the effects are short term. Other studies find very limited changes following lifestyle related diagnosis such as diabetes, heart disease, high blood pressure or high cholesterol (Hut and Oster 2022; Rahkovsky, Anekwe and Gregory 2018). Studies on the effects on diet following diabetes type 2 suggest small improvements in overall dietary healthiness that decline over time (Edenbrandt et al. 2022; Oster 2018) or no effects in fruit and vegetable consumption (Thomas and Mentzakis 2024). Notably, although there is evidence of specific negative health shocks affecting diets, there is little evidence on how the strength of the negative health shock affect behavioral response. While Hut and Oster (2022) include both severe (diabetes and heart disease) and milder (high blood pressure, high cholesterol) health shocks in their analysis on the effects on food purchases, they do not investigate the behavioral response for the different types of diagnosis separately. An exception to this is Verdun (2020), who uses longitudinal data on UK individuals and find that those diagnosed with a severe health shock (diabetes or heart attack) improved their diet considerably, while those experiencing a mild health shock (angina or high blood pressure) did not change food consumption patterns significantly. However, this study does not examine the interaction effect of these health shocks.

The contribution of this study is twofold. First, we investigate whether individuals diagnosed with lifestyle related conditions adjust their food consumption in accordance with the dietary recommendations given in relation to the diagnosis. We investigate both immediate and longer‐term effects following a negative health shock. While previous research in the field of health economics explores the behavioral response to lifestyle related diagnosis, our study provides insights into how the severity of such diagnoses influence food choices. We examine whether changes in food consumption following a mild health shock (high cholesterol or high blood pressure) differ from those following a severe health shock (a CVD). Furthermore, we analyze whether self‐selection occurs, such that individuals who previously did not respond to a mild health shock fail to respond to a severe health shock. Overlooking such self‐selection could lead to misinterpretation of the results when comparing the dietary effects of mild versus severe health shocks.

While our first contribution is an expansion of existing literature in the field, our second primary contribution is that, compared to previous studies, our identification of the timing of diagnoses is highly reliable and precise. We use several linked data sets, including official register data containing high quality information about diagnosis, hospitalization and medical purchase data, matched with consumer panel data, wherein individuals register their food purchases on a daily basis. Prior research typically relies on self‐reported diagnosis status for health and/or food consumption. For example, Hut and Oster (2022) use self‐reported diagnosis of diabetes, heart disease, high cholesterol and high blood pressure, with information on the year of diagnosis. Other studies also use self‐reported data on diagnosis (Kim et al. 2018; Rahkovsky, Anekwe, and Gregory 2018; Thomas and Mentzakis 2024; Verdun 2020) or self‐reported food consumption data (Kim et al. 2018; Thomas and Mentzakis 2024; Verdun 2020; Zhao, Konishi, and Glewwe 2013), which may raise concerns of reliability and/or with measurement errors.

The remainder of this paper is organized as follows: Section 2 presents the theoretical framework used to test the research questions regarding effects from negative heath shocks. Section 3 presents the data and summary statistics, while Section 4 presents the empirical framework including the identification strategy. Section 5 presents and discusses the results, followed by a set of sensitivity analysis. Finally, Section 6 concludes and suggests directions for future research.

## 2 Theoretical Framework

We take outset in a simple model of food consumption (*y*), allowing mild and severe health shocks (MHS and SHS) to affect various elements of food consumption:

$$y=\beta_{0}+\beta_{1}\text{MHS}+\beta_{2}\text{SHS}+\beta_{3}\text{MHS}*\text{SHS}$$

We hypothesis that food consumption shifts in the direction of increased healthiness following both mild and severe health shocks. Thus, if *y* is a measure of the healthiness of the diet, we expect $\beta_{\text{MHS}}>0$ and $\beta_{\text{SHS}}>0$ as previous empirical studies provide weak support for improved healthiness in dietary patterns following both MHS and SHS (Carrera, Hasan, and Prina 2020; Dai et al. 2022; Hut and Oster 2022; Rahkovsky, Anekwe, and Gregory 2018; Verdun 2020; Zhao, Konishi, and Glewwe 2013), although others find no significant effects (Thomas and Mentzakis 2024).

The risk of severe negative health outcomes is larger following a SHS compared to a MHS. Theoretically, this would suggest that the shift in food consumption toward increased healthiness is stronger following a SHS, such that $\beta_{\text{SHS}}>\beta_{\text{MHS}}$. However, this expectation ignores the possible self‐selection bias, which occurs if individuals that have failed to react to previous negative health signals, including MHS, disproportionally represent individuals that are diagnosed with SHS. For this reason, we expect that individuals that get a SHS and that did get a MHS previously react less from the SHS, since they have previously failed to react to warning signals. Thus, we expect that $\beta_{\text{MHS}*\text{SHS}}<0$.

We note that a potential effect in the opposite direction to this prediction can occur; suppose that some individuals suffer from HA or HC, but do not visit a doctor or hospital to have it diagnosed or treated. These individuals will appear as not having had a warning (MHS) prior to the SHS, but in fact they suffer from a mild health shock diagnosis but are ignorant about treating it.

## 3 Materials

Our analysis is based on multiple sources of data that are merged on the individual level: (1) food purchase data concatenated with (2) nutrient data, (3) individual data on health shocks in the form of cardiovascular disease (CVD) and arterial hypertension (HA) and hypercholesterolemia (HC), and (4) individual socio‐demographic data.

### 3.1 Food Purchases and Nutrient Content of Foods

An individual's food consumption is measured based on food purchases by the household. Food purchase data is provided by GfK ConsumerScan and our data period runs over the period 2006–2017. The data set consists of observed food purchases by a panel of households in Denmark that register all their food purchases with a home scanner device, providing information about the purchases of all food products on the UCP level. During the period included in the study, there were in total 8524 households (16395 individuals) that registered their purchases for at least some period. The dataset is unbalanced, such that some households only report for a few months while other households reports for several years. The panel provider seeks to hold a representative sample of Danish households with respect to age, education, family size and geographical location, and households that exit the panel are replaced by households with similar demographic characteristics.

The purchase data from the consumer panel is linked with nutrient content data based on the Danish Food Composition Databank managed and updated by the National Food Institute (2019). This provides data on the content of energy and macronutrients (such as carbohydrates, protein and fat) and sub‐categories of macronutrients (such as sugar, fiber, saturated fat) content per 100 g of each of the food products in the databank. There were 1049 different food products in the database at the time of establishment of the dataset, and for foods that are not included in the databank, the nutritional content for that food is approximated based on an average recipe for the food.

While the food purchase data is provided on household level, we want to include individuals' food purchases in the analysis. We thus construct individual consumption for each member of the household, were each household member is given a weight relative to the gender and age dependent recommended daily energy intake based on the Danish National Survey of Diet and Physical Activity in Denmark.^1^ We use two main types of measures for dietary quality, which are selected based on the specific dietary recommendations that are given to individuals diagnosed with CVD, HA or HC respectively. First, to analyze if the overall healthiness of an individual's food consumption changes following a diet, we use a composite measure of the healthiness of the diet in the form of a Healthy Eating Index (HEI). The HEI measure is constructed similar to the healthy eating index presented in Gil, de Victoria, and Olza (2015), but aligned to the recommendations provided by the Danish Ministry of Family and Consumer Affairs. The HEI therefore evaluates the adherence to the official dietary guidelines. The HEI gives a measure of the dietary healthiness of the household covering eight aspects of the diet including the amount of fruit and vegetables, fish and the content of sugar, fat and fiber. The HEI ranges from 0–100, where 100 implies perfect adherence to the dietary recommendations. In the data, the average HEI is 76, with a standard deviation of 7, minimum is 44 and maximum 99. More details on how the HEI is computed is available in Section S1.

Second, we use specific nutrients and food categories as measures of dietary quality. The two key nutrient groups considered are saturated fat and fiber, of which both are key elements in a diet aimed at reducing the risk of getting CVD based upon a diagnosis of HA or HC or reoccurrence of CVD complications. The variables are included as energy percentages, and they are calculated as grams of the nutrient purchased times the energy density in the nutrient in kJ/g and then divided by total energy consumption for the individual. Moreover, we include three food categories: fruit and vegetables, fish and red meat. Each of these are selected based on the recommendations for dietary changes to individuals with CVD, HA or HC. We note that several more refined dietary shifts within a food category could be relevant to consider. For example, for dairy, the recommendations are to substitute from high fat dairy and toward low fat dairy.

We calculate each of the dietary measures on a monthly basis. While the data on food purchases is considered of high quality, consumption away from home, such as restaurants and canteens, are not included. The share of out of home consumption is relatively low in Denmark, and for most individuals it is considered a luxury. Eating out and takeaway food constitutes around 9% of the dinners, and it is mainly the younger age groups (below 30 years) that eat out more frequently (Madkultur 2021). The main part of lunches are also brought from home, where 72 to 91% of the respondents in the GfK consumer panel indicate that they eat lunch prepared at home on an average week, with the lower share for working adults and children in school and higher share for retired and unemployed. Hence, we consider food consumed at home to be reasonable for representing the dietary patterns for the population that we consider in this analysis.

### 3.2 Identification of Occurrence of Health Shocks

For all individuals in the households that participated in the consumer panel during the period of this study, we obtain high quality register data regarding health shocks related to CVD, HA and HC as well as type 2 diabetes (T2D) that we use as a control since diagnosis of diabetes type 2 is also associated with dietary recommendations that may affect food choices.^2^ For each of these health shocks, we obtain information from two sources. First, we obtain information about when an individual has been hospitalized and registered with a treatment or diagnosis for CVD, HA, HC or T2D. These data are obtained from the national patient register, and the date of the complication is registered. The first occurrence of a hospitalization or treatment for an individual is identified as the time of the health shock. Second, we obtain information about when an individual has been prescribed and purchased medication used for treatment of CVD, HA, HC or T2D. Information about the purchase of these prescribed medications is provided by the Danish Medicines Agency. The first occurrence of a medication prescription is used as identification of health shock. A full list of the complications as registered at hospitals, and the medication prescriptions used for identification of CVD, HA and HC is provided in Section S2.

A limitation with the data is that it does not include diagnosis made by general practitioners (GP), although the GPs' medical prescriptions are included. Yet, individuals that are diagnosed by a GP, and neither are treated for any complication nor prescribed any medicine, are not identified as individuals with CVD, HCP, HB or T2D in our sample. We expect that this might be the case for MHS such as HA and HC, while GPs do not diagnose CVD without further investigation or medication. Thus, if anything, we underestimate the dietary effects following a MHS.

### 3.3 Personal Characteristics

All individuals in the households that participate in the consumer panel during the period of this study are linked with registry data from Statistics Denmark, providing information about family composition, income, education level and public support.

The linking of the different data sets was conducted by Statistics Denmark, and any personal identifiers (personal identity number, names and addresses) of the individuals were replaced by anonymous unique identification numbers in the data set that we access. Moreover, data are only available through the servers of Statistics Denmark, and only estimation results and average descriptive statistics can be exported from the server.

### 3.4 Descriptive Statistics

To achieve the objectives of this study, we focus on individuals diagnosed while participating in the consumer panel and reporting their food purchases. Thus, any individual who experience a health shock during a period when they are not reporting food purchases is excluded from further analysis. We recognize that in a period of hospitalization, there may be some disruptions in reporting to the consumer panel, so to be part of the sample used in the analysis diagnosed individual must have reported food purchases in at least one of the 3 months preceding a health shock, and in at least one of the 3 months following the health shock.

We identify four possible scenarios that individuals can encounter during our data period: (a) no health shocks (control group) (b) SHS while reporting to panel (c) MHS while reporting to panel and (d) first MHS and then later a SHS while reporting to panel. Individuals that get SHS or MHS prior to reporting are excluded. For individuals that get MHS and SHS in same month (while reporting), MHS is set to zero, since the SHS is the strongest shock and will dominate, and they are thus included in group b. Individuals that encounter a health shock in the first month of reporting food purchases in the panel are excluded for the reasons mentioned above. Individuals with no health shocks that are included in a household with an individual in scenario b, c or d are excluded from the control group. A summary is presented in Table 1.

**TABLE 1:** Sample overview.

	Scenarios for individuals			
	A	b	c	d
MHS while reporting	0	0	1	1
SHS while reporting	0	1	0	1
Individuals	9672	119	925	107

In total, there are 9672 individuals that have no diagnosis with CVD, HA or HC before or during their participation in the panel. These are included as a control group. There are 1151 individuals that get a health shock while participating in the panel, and this includes both CVD, HA and HC. Descriptive statistics for the sample are presented in Table 2. The treated samples consist of older individuals, and this is reflected in the lower household size and with fewer children living at home. There are also more single households. Summary statistics for the samples used for sensitivity and robustness analysis are presented in Section S3, Tables S3.1—S3.3. There are significant differences in the purchase patterns between the control group and the treated. In these comparisons, only purchases in the months prior to the health shocks are included, to exclude any effect on purchases following the diagnosis. The treated samples have somewhat higher overall healthiness (HEI), but while the difference is statistically significant, the magnitude is small. Focusing on the key nutrients and product categories where high levels of consumption are related to CVD, HA and HC, we see that the share of saturated fat is significantly higher among the treated, and this is in line with the main risk factors for CVD, HA and HC. The treated have a significantly lower consumption of fruit and vegetables. In the SHS + MHS‐group, fruit and vegetables comprise 5.8% of their total energy consumption compared to 8.3% for the control group. The treated samples have higher consumption of red meat with, where the average in the three treated samples range from 11.3 to 12.7% of their total energy consumption compared to 10.3% for non‐treated. The patterns are the same for the sample of single households, although the differences for HEI and fruit and vegetables are not statistically significant.

**TABLE 2:** Descriptive statistics for control sample and treated samples.

	Control	SHS‐group	MHS‐group	MHS + SHS‐group	Test for difference across groups (*p*‐value)
Personal or household characteristics					
# Individuals	9672	119	925	107	
Female (%)	51.96	44.54	58.59	43.93	< 0.001
Age (mean)	38.51	63.36	64.03	68.30	< 0.001
Household size (mean)	2.71	1.97	1.89	1.81	< 0.001
Single household (%)	15.85	27.73	30.27	31.78	< 0.001
Income in DKK (mean)	255,881	249,697	255,659	227,400	0.444
Type 2 diabetes (%)	1.26	10.08	19.35	22.43	< 0.001
Food purchase patterns					
HEI	76.25	77.69	77.48	78.08	< 0.001
Saturated fat (E%)	14.77	15.79	15.85	15.71	< 0.001
Added sugar (E%)	5.57	4.41	4.24	3.87	< 0.001
Fiber (E%)	2.17	2.31	2.20	2.26	0.492
Fruit and vegetables (E%)	8.43	7.02	6.76	5.84	0.001
Fish (E%)	0.96	1.25	1.62	1.59	< 0.001
Meat (E%)	10.33	11.94	12.68	11.28	< 0.001
Sugar‐sweetened beverage (E%)	2.65	1.51	1.84	1.83	< 0.001

*Note:* For age and income there are missing values in the control group, resulting in that average age is calculated using 8449 observations and average income is calculated using 9659 observations. For the food purchase patterns, the treated group only includes observations prior to the treatment. The calculation of the E%‘s is explained in Section S1.

While not a key aspect for reducing the risk of CVD, HA or HC, we present the energy share from added sugar and a food category high in added sugar (sugar‐sweetened beverages). For all three treatment groups, the sugar levels and SSB are significantly lower compared to the non‐treated. The HEI is calculated based on eight aspects, one of which is added sugar (Section S1). The calculation of added sugar includes all product categories, including SSBs. Thus, the higher consumption of added sugar, including sugar from SSBs, in the control group explains the relatively small differences in the final HEI scores between the control and treatment groups. If we only consider the factors that are key elements for developing HA, HC and CVD our treatment groups are considered to have a less healthy diet compared to the control group.

## 4 Empirical Framework

We use the panel structure of the data and apply a difference‐in‐differences analysis. We test for the effects of health shock on several different diet‐related measures that are key elements in the recommendations provided for individuals when diagnosed with CVD, HA or HC; HEI, saturated fat, fiber, fruit and vegetables, fish and red meat. In the following two‐way fixed effects (TWFE) model, $y_{i,t}$ represents the different diet‐related measures for individual *i* at time *t*:

(1) $$y_{i,t}=\beta_{0}+\beta_{1}{\text{MHS}}_{i,t}+\beta_{2}{\text{MHS}}_{i,t-3}+\beta_{3}{\text{MHS}}_{i,t-6}+\beta_{4}{\text{MHS}}_{i,t+3}+\;\beta_{5}{\text{SHS}}_{i,t}+\beta_{6}{\text{SHS}}_{i,t-3}+\beta_{7}{\text{SHS}}_{i,t-6}+\beta_{8}{\text{SHS}}_{i,t+3}+\;\beta_{9}{\text{SHS}}_{i,t}*{\text{MHS}}_{i,t}+\beta_{10}{\text{SHS}}_{i,t-3}*{\text{MHS}}_{i,t}+\beta_{11}{\text{SHS}}_{i,t-6}*{\text{MHS}}_{i,t}+\;\beta_{12}{T2D}_{i,t}+\beta_{13}{T2D}_{i,t-3}+\beta_{14}{T2D}_{i,t-6}+\beta_{15}{T2D}_{i,t+3}+\sum_{j=1}^{11}\partial_{j}{\text{month}}_{j}++\sum_{k=1}^{11}{\partial \gamma }_{k}{\text{year}}_{k}+a_{i}+\varepsilon_{it}$$

The mild health shock (MHS) is a dummy variable, taking the value one from the month an individual had a MHS and onwards, and zero otherwise. The same holds for the severe health shock (SHS) variable. The immediate effects from the health shocks are captured in $\beta_{1}$ and $\beta_{5}$.

To test for longer‐term effects following a health shock, we include 3‐month and 6‐month lags ($\beta_{2}$ and $\beta_{3}$ for MHS and $\beta_{6}$ and $\beta_{7}$ for SHS). The lagged treatment variables are constructed as dummy variables, taking the value of one from the month an individual experiences a health shock and for the following three (six) months. The variables are zero otherwise. For CVDs treated at the hospital, there is typically a follow‐up session with the medical doctor and/or cardiac rehabilitation nurse within one‐two months after the hospitalization, where, among other things, dietary advice is provided. For this reason, we expect a further improvement in healthiness in diet at this point in time, in the form of a positive effect on the 3‐month lag for the SHS. For the unhealthy outcomes (red meat and saturated fat) we expect the opposite signs.

We include lead‐variables, which takes the value one 3 months prior to the actual month of the health shock and 0 otherwise. This implies that the coefficients on the lead variables $\beta_{4}$ and $\beta_{8}$ will test if individuals change dietary behavior prior to the health shocks.

To test if the strength of the health shock affects the behavioral response, we want to compare the effects from SHS and MHS, while controlling for if the individuals with a SHS had a warning in terms of a MHS prior to the SHS. We want to test if the effects from a SHS are different for individuals that have obtained a MHS prior to their SHS, and thus include interactions ($\beta_{9},\beta_{10}$ and $\beta_{11}$). These interactions will test for whether there is a self‐selection effect in the SHS.

Finally, we include a set of control variables. First, we include variables for diabetes type 2 diagnosis (T2D), as this is expected to affect diets. We include these variables in the same form as the SHS and MHS, allowing for longer‐term (lagged) effects and lead variables. Second, we include year ($\partial_{k})$ and month $\left(\partial_{j}\right.$) fixed effects, to control for seasonal effects and for longer term changes in food purchases over the time span of the data, leaving out January as the base month. The term *a*_*i*_ is an individual fixed effect and $\varepsilon_{it}$ are regression disturbances with mean zero that are independently distributed. To accommodate for general forms of autocorrelation in $\varepsilon_{it}$ across months for an individual, and that this pattern may differ across individuals, we report test statistics based on standard errors that are robust to heteroscedasticity and autocorrelation and clustered at the individual level.

### 4.1 Heterogeneity Analysis

We conduct a set of heterogeneity analyses to investigate the extent to which the reaction in the dietary response to MHS and SHS varies across the sample. First, we descriptively examine the distribution of changes in dietary healthiness following SHS and MHS. Second, we investigate if the dietary quality prior to treatment correlates with the change in the dietary outcome following the treatment. For example, we explore if individuals with very healthy diets improve more or less following a treatment. To do this, we calculate each individual's change in dietary outcome (${\overset{∼}{y}}_{i}$) as the difference between the average dietary quality in the 3 months following treatment and the average in the 3 months preceding it $\left({\overset{∼}{y}}_{i}={\bar{y}}_{i,\text{post}-\text{treatment}}-{\bar{y}}_{i,\text{pre}-\text{treatm}ent}\right)$. We regress this variable on the dietary quality in the months prior to treatment:

(2) $${\overset{∼}{y}}_{i}=\beta_{0}+\beta_{1}{{\bar{y}}_{i,\text{pre}-\text{treatment}}}_{i}+\varepsilon_{i}$$

We first use ordinary least squares regressions to analyze average differences, and then use quantile regressions to examine the relationship between these variables at different quantiles of the distribution of dietary quality. In these analyses, only the treated individuals are included.

### 4.2 Robustness Analysis

To check the robustness of our analysis we do sets of sensitivity analyses. First, the food purchase data is reported on the household level, hence if the diet of one individual in the household changes, while the remaining household members maintain their diet, the changes made by the diagnosed individuals will be dampened in our data. For this reason, dietary changes can be expected to be stronger when only including single households. However, there is evidence that individuals that have a partner are more likely to participate in supportive information sessions, and to medicate according to their prescriptions, which might counteract the above mentioned effect (Doherty et al. 1983; Wu et al. 2012). We do thus not know a priori if the effects from health shocks are different among single households compared to the full sample. Furthermore, the effects of MHS and SHS that we estimate might be influenced by changing composition of the households. We therefore estimate a model where we only include individuals that live in households with a constant number of individuals throughout the reporting period, thus eliminating any potential effects in the full sample from changes in household composition.

We noted in the section on descriptive statistics for the full sample that the treated groups are notably older than the control group, and this is to be expected with the higher prevalence of CVD, HA and HC in higher age groups. If we restrict the sample to include only individuals above 55 years, the difference in observable characteristics between the control and treated groups declines (see descriptives of this sample in Table S3.3). As a robustness analysis we therefore re‐estimate the models including only individuals aged 55 and above.

A potential concern with TWFE analysis involving staggered treatment is that estimates can be biased if treatment effects are heterogenous (Baker, Larcker and Wang 2022). One source of bias arises when the effect from a health shock varies over time, leading individuals treated early on to serve as an inappropriate comparisons for those treated later (Roth et al. 2023). In our data set, some individuals remain in the panel many months post‐treatment, while others exit shortly after. Recent advances in econometrics propose estimators for TWFE with staggered treatments (Baker, Larcker, and Wang 2022). However, when multiple treatments are involved, methods to address such biases are still under development. One recent study offers a method for settings with multiple treatments but this assumes that all individuals who receive a second treatment have previously undergone the first treatment (de Chaisemartin and D’Haultfœuille 2023). This assumption does not hold in our case, where we aim to examine differences in treatment effects based on whether those in the second treatment had received the first. To assess whether individuals treated early, with many post‐treatment observations, influence the estimated treatment effects, we re‐estimate the models, excluding observations after 6 months or more after the treatment (for individuals in the MHS + SHS we exclude observations 6 months or more after the SHS treatment).

The data used consists of an unbalanced panel, where the length of participating in the panel varies between individuals. When a household leaves the panel, they are replaced by an individual with similar characteristics to maintain the socio‐demographic composition of the panel. Few individuals report over the whole period included in our data, so almost everybody leaves the panel at some point. This is a concern if the point of leaving the panel is correlated with the behavioral response to a treatment. Say that individuals that react in a specific way to a treatment are more likely to leave the panel prior to the treatment, this may bias our results. We do not have data on individuals after leaving the panel, so we resort to examining the patterns for length of reporting. Individuals in the control group report fewer months on average compared to those in the treated groups (Table S3.4). The participation time is negatively related to age, and when only including individuals above 55 years, the differences decline. Importantly, those in the treated groups will by design stay longer in the panel since they must stay long enough in the panel to report both before and after a treatment, particularly those in treatment MHS + SHS, since they must stay long enough to report both before and after a MHS and at a later point a SHS. We split each treatment group on the median number of months of reporting to the panel and estimate the model for these two groups separately to test this effect.

## 5 Results and Discussion

### 5.1 Effects From Mild Health Shocks

Table 3 presents the results from the regression models (Equation 1). All hypothesis tests are one‐sided, and we focus on tests with a significance level of 10% or lower. In line with prior expectations, there are immediate improvements in healthiness following a MHS for all dietary outcomes, although the effects are only statistically significant in one‐sided tests for HEI and fish ($H_{0}:\beta_{\text{MHS}}=0,H_{1}:\beta_{\text{MHS}}>0)$. For most of the dietary measures, the immediate dietary improvements are followed by declines in healthiness in the longer term. This is indicated with negative parameters values for MHS_−3_ and MHS_−6_. For HEI, there is an immediate increase of 0.55, a decline of −0.32 after 3 months and a further decline of −0.13 after 6 months, and the total (aggregated) effect is statistically not different from zero (Test MHS^,^ bottom panel in Table 3). For fish, this pattern is similar, where the initial increase is followed by a decline after 3 months and where the total effect is not different from zero. For none of the models are there any statistically significant difference for the overall improvement in healthiness (decline in consumption).

**TABLE 3:** Effect of health shocks on dietary patterns.

	HEI	F&V	Fish	Red meat	Sat fat	Fiber
Mild health shock effects						
MHS	0.55***	0.11	0.18**	−0.32	−0.16	0.01
	(2.38)	(0.68)	(1.69)	(1.16)	(1.20)	(0.59)
MHS_−3_	−0.32*	−0.01	−0.33***	0.01	0.03	0.00
	(1.48)	(0.05)	(3.62)	(0.06)	(0.20)	(0.15)
MHS_−6_	−0.13	−0.34***	0.08*	−0.10	0.12	−0.03*
	(0.72)	(2.33)	(1.33)	(0.51)	(1.19)	(1.54)
Strong health shock effects and interactions						
SHS	0.77*	1.04**	0.14	−0.57	−0.46*	0.16***
	(1.44)	(2.30)	(0.74)	(0.99)	(1.33)	(2.39)
SHS_−3_	0.95**	−0.76*	0.11	−0.38	−0.48*	−0.05
	(1.77)	(1.40)	(0.40)	(0.65)	(1.52)	(0.76)
SHS_−6_	−1.17***	0.73*	−0.15	0.56	0.44*	−0.13***
	(2.66)	(1.46)	(0.81)	(1.24)	(1.59)	(2.38)
SHS × MHS	−1.28**	−0.89*	−0.06	2.11***	0.71*	−0.18**
	(1.75)	(1.51)	(0.20)	(2.96)	(1.62)	(2.14)
SHS_−3_ × MHS	−0.27	0.79	−0.08	−1.04	0.51	0.08
	(0.34)	(1.26)	(0.24)	(1.14)	(1.08)	(0.80)
SHS_−6_ × MHS	0.73	−0.67	0.15	−0.32	−0.35	0.11*
	(1.16)	(1.11)	(0.65)	(0.53)	(0.90)	(1.36)
Control variables						
T2D	1.83***	1.25**	0.34*	−1.29**	−0.71**	0.12**
	(3.21)	(1.92)	(1.59)	(1.65)	(2.13)	(1.74)
T2D_−3_	0.02	−0.27	−0.15	−0.40	0.05	−0.01
	(0.04)	(0.87)	(0.75)	(0.62)	(0.18)	(0.21)
T2D_−6_	−1.46***	−0.37	−0.20	0.84**	0.57***	−0.09**
	(3.78)	(0.74)	(1.22)	(1.94)	(2.36)	(2.14)
Lead variables						
MHS_+3_	0.22	0.23*	0.04	0.09	−0.13	0.03*
	(1.12)	(1.38)	(0.45)	(0.44)	(1.09)	(1.64)
SHS_+3_	0.42	0.36	0.15	−0.09	−0.08	0.01
	(1.40)	(1.19)	(1.22)	(0.24)	(0.41)	(0.19)
T2D_+3_	−0.65*	0.20	−0.22*	0.51	0.05	0.00
	(1.44)	(0.32)	(1.36)	(0.90)	(0.20)	(0.02)
Intercept	79.66***	7.48***	1.39***	11.64***	14.63***	2.40***
	(686.37)	(77.77)	(34.10)	(99.30)	(214.57)	(200.32)
Within *R* ^2^	0.02	0.01	0.01	0.01	0.02	0.02
*F*‐statistic	77.47	50.45	22.01	16.55	82.70	96.55
Test MHS^a^ (*p*‐value)	0.656	0.175	0.469	0.102	0.914	0.608
Test SHS^b^ (*p*‐value)	0.211	0.069	0.674	0.371	0.071	0.769

*Note:* |*t*‐values| in parenthesis. *p*‐values for one‐sides tests are indicated by *p < 0.10, **p < 0.05, ***p < 0.01. Year and month fixed effects included in all models. *N* = 262761 individuals = 4780.

^a^ MHS + MHS_−3_ + MHS_−6_ = 0.

^b^ SHS + SHS_−3_ + SHS_−6_ = 0.

### 5.2 Effects From Strong Health Shock

The immediate effects following a SHS also imply improvements in healthiness, and the effects are statistically significant in one‐sided tests for HEI, fruit and vegetables, saturated fats and fiber ($H_{0}:\beta_{\text{SHS}}=0,H_{1}:\beta_{\text{SHS}}>0)$. For several of the outcomes (HEI, saturated fat) the healthiness improves further after 3 months. We expect that this is explained by the follow‐up consultation that is offered approximately one to 2 months after the complication is treated at the hospital. However, 3 months after the follow‐up consultation (SHS_+6_), the effects are reversed for several of the outcomes (HEI, saturated fat, fiber). For fruit and vegetables, the decline occurs after 3 months followed by an improvement after 6 months—a pattern that stand out compared to the other categories, and for which the reasons remain unclear. Interestingly, contrary to the MHS, the total effects after 6 months suggest improvements, and these are statistically significantly different from zero for saturated fat and fruit and vegetables.

Comparing the effects for mild and strong health shocks, we see that for HEI, saturated fat, fiber, fruit and vegetables and red meat, the size of the effect is larger for the SHS and it is more persistent over time. Thus, the prediction of stronger effects following more severe health shocks is supported, and this is also in line with findings in Verdun (2020).

In line with expectations, the interaction between SHS and MHS is negative immediately following the health shock (and positive for the unhealthy outcomes saturated fats and red meat). This suggests that individuals that have had a MHS prior to their SHS change their diet less after the SHS or not at all or even in an unhealthy direction. These effects are statistically significant in one‐sided tests for HEI, fruit and vegetables, red meat, saturated fats and fiber ($H_{0}:\beta_{\text{MHS}*\text{SHS}}=0,H_{1}:\beta_{\text{MHS}*\text{SHS}}<0)$.

Hence, those that have had a previous health shock adjust their diet less following a more severe health shock. This may indicate the existence of a self‐selection bias. For those that did not have a previous health shock, the effect sizes are larger for the stronger health shock than the mild health shock. This holds for HEI, fruit and vegetables, saturated fat and fiber.

Our main hypothesis is (1) that MHS has a smaller effect than SHS on dietary improvements, and (2) that there might be self‐selection bias in whom gets a SHS. Thus, if we want to compare the effect on dietary behavior from MHS and SHS we should control for such self‐selection bias. We include results from a naïve model specification where we fail to control for this (Table S3.5). As expected from our hypothesis, the conclusions from this alternative specification are different, suggesting that the SHS is not associated with a stronger behavioral response compared to MHS. Hence, without accounting for the self‐selection into SHS might lead to a bias in the estimated effects of health shocks on dietary healthiness.

### 5.3 Control Variables

We include lead variables that measure if there are dietary changes 3 months prior to the heath shock. This represents an artificial treatment 3 months prior to the actual treatment. We do not find any of these leads statistically significant, hence there are no changes in dietary healthiness indicating that the individuals do not have expectations of a treatment.

The overall pattern for the variables controlling for a T2D diagnosis follows that of MHS and SHS with immediate improvements in the diet (HEI, fruit and vegetables and fiber and decreased intake of saturated fats), followed by a stagnation and then declines in healthiness after 6 months.

### 5.4 Treatment Heterogeneity

There is substantial heterogeneity in the change in dietary outcome following the treatments. If we focus on the overall healthiness of diets, HEI, the median change following a MHS is −0.10, while the 95th percentile improve by 8.19 the 5th percentile decrease HEI by 8.17. Numbers are similar following an SHS (descriptive statistics are presented in Table S3.6). Regressing the dietary quality prior to the treatment on the change in the dietary outcome following a health shock shows that a healthier diet is associated with lower changes following a health shock (results are shown in Table S3.7). We also examine whether the correlation with pre‐treatment dietary quality varies with dietary changes using quantile regressions. The pre‐treatment dietary quality is negatively correlated with dietary change across all quantiles, with a stronger effect observed in the higher quantiles (Table S3.8 and Figures S3.1 and S.3.2). A potential explanation could be that those with a healthier diet prior to a health shock have less room for improvement. Further, as noted by a reviewer, the health shocks studied are not only caused by unhealthy diets but are also related to genetics and other life‐style related factors, such as smoking and physical activity. Although we lack data on these factors, heterogeneity in dietary changes may correlate with other lifestyle adjustments. For instance, individuals with healthier pre‐treatment diets might focus on improvements in other life‐style factors.

### 5.5 Robustness Analysis

We re‐estimate the models presented in Equation (1) while only including individuals that are single households. Descriptive statistics are available in Table S3.2, and comparison with the full sample (Table 2) shows that the single household sample has a larger share of females and the consumption in the control group is lower in meat and higher in added sugar compared to the full sample. Results for the models for the single household sample are presented in Table S3.9. Overall, the patterns found for the full sample remains. In both samples, we find an immediate improvement in HEI following a SHS, and this continues to improve after 3 months, while the trend turns after 6 months. The effect sizes are distinctly larger in the sample with singles only. Individuals in single households that had a MHS prior to a SHS reduce their HEI immediately following the SHS. Interestingly, following the immediate decline in HEI they maintain this level in the longer run, and do thus not decrease dietary quality after 6 months like those without a prior MHS do. The patterns observed for the HEI are similar for red meat, saturated fat and fiber. The size of our sample for singles prevents us from exploring these effects in more detail, but we note that this is a potential area for future research.

One reason for the larger in magnitude effects in the model including only single household could be that the non‐treated individuals in the same household do not change behavior, at least not to the same extent as the treated. The effects on the treated are then diluted. Another potential reason is household composition changes, where individuals with certain food preferences leave or enter a household and affect the average consumption. We estimate a model where we only include individuals in households with a constant number of individuals throughout the reporting period, thus eliminating any potential effects in the full sample from changes in household composition. Results are presented in Table S3.10. Compared to the results for the full sample, presented in Table 3, the main results are the same, while the statistically significant coefficients are larger in most models where the household composition is constant. For fruit and vegetables as well as saturated fat there is a statistically significant improvement after 6 months. These results suggest that part of the difference between the full sample and the sample with only singles is related to the entry and exit of household members, while part of it is related to the diluting effect from non‐treated individuals in these households.

To account for the differences in the descriptive statistics for the control and treatment groups as observed in Table 1 we also re‐estimate the models including only individuals aged 55 and above as a robustness test (Table S3.11). This reduces both the total number of observations and the number of treated individuals, though it primarily reduces the size of the control group. Compared to the full sample results in Table 3, the effects are smaller in magnitude, with fewer significant coefficients, which might be due to the reduced sample size.

As explained in the empirical framework we re‐estimate the models, excluding observations 6 months or more after the treatment (for individuals in the MHS + SHS we exclude observations 6 months or more after the SHS treatment) to assess whether individuals treated early, with many post‐treatment observations, influence the estimated treatment effects. Results are presented in Table S3.12. Treatment effects are very similar to those estimated from the full sample. An important difference in these models is that the total effect from SHS is statistically significant for HEI, red meat, saturated fat and fiber, but we note that this is likely explained by that the 6‐month MHS lag drops out.

To explore concerns with attrition, we split each treatment group on the median based on number of months of reporting to the panel and estimate the model for these two groups separately (Table S3.13). Comparisons do not suggest important differences, as the total effect for each treatment is similar in the below median and above median groups. The timing of the effect varies somewhat, as seen in the SHS‐group models where the above median model shows a strong immediate positive effect, while the below median group has a similar effect after 3 months. It should be noted that the number of individuals in each group, when divided by the median, is relatively low, particularly for the SHS group.

## 6 Conclusions

We investigate the effects of severe health shocks on food consumption. Our study utilizes multiple high‐quality datasets, providing precise and reliable information on various health shock occurrences such as cardiovascular disease (CVD), arterial hypertension (HA, measured by blood pressure) and hypercholesterolemia (HC, measured by cholesterol levels), as well as T2D, as reported in official statistics from Statistics Denmark. Additionally, we access data on medication prescriptions. These sources enable us to identify the timing and occurrence of these health shocks, which we then combine with food purchase data obtained from a consumer panel. To enhance the food purchase data, we include information on the nutrients content in each product purchased, allowing us to construct measures of overall healthiness and the proportion of energy derived from specific nutrient and food categories. Previous findings are based on self‐reported diagnosis, typically with imprecise time of diagnosis (Kim et al. 2018; Rahkovsky, Anekwe, and Gregory 2018; Verdun 2020; Thomas and Mentzakis 2024, Hut and Oster 2022) and/or self‐reported food consumption data (Kim et al. 2018; Thomas and Mentzakis 2024; Verdun 2020; Zhao, Konishi, and Glewwe 2013), which may raise concerns on reliability and/or with measurement errors.

Our findings indicate that dietary responses occur following both severe heath shocks (SHS) and mild health shocks (MHS), characterized by initial improvements that decline over time. For overall diet quality, measured by a healthy eating index, as well as for specific food groups and nutrients, the overall treatment effects after 6 months are not different from zero. This underscores the challenges of sustaining dietary improvements over the long term.

An important discovery of our study is the presence of self‐selection bias when examining the effects of SHS. Both a MHS and a SHS leads to immediate positive dietary changes, with a stronger effect of a SHS than a MHS. However, for individuals that are exposed to both shocks the effect from SHS is close to zero, indicating that these individuals failed to react to the MHS and also fail to react to the SHS. Failing to account for this bias may lead to the misconception that severe health shocks do not result in dietary improvements. We conclude that individuals who experience a SHS without prior mild health issues (high blood pressure or high cholesterol) exhibit significant behavioral shifts. However, among those who have previously experienced MHS, there is minimal to no effect on food consumption following a SHS. This suggests that the severity of the health shock corresponds to the strength of the behavioral response when it is the individual's first experience of such a shock.

The sensitivity analysis based on single households show that the longer‐term effects remain statistically insignificant, but the short‐term effects are stronger than for the full sample. We note that this may be driven by (i) that any dietary behavioral changes following a health shock made by the individual is dampened in our data if the other household members do not make such changes, since our purchase data is reported on household level. (ii) Other household members may encourage behavioral change if a household member receives a health shock. Such encouragement effects have been found in (Jeemon et al. 2021; Vedanthan et al. 2016). Our analysis suggests that the dampening effect is larger than the encouragement effect, but our sample size for single households is not large enough to explore this in more detail. Such analysis on household member effects on behavioral change is an interesting venue for future research.

It is important to note that while our data is of high quality and detail, individuals may experience MHS as warning signals for CVD beyond HA and HC. Therefore, even individuals identified in our study as having had no prior warning signals (no mild health shock) may indeed have received warnings. Unfortunately, our data does not allow us to control for such signals. A further limitation with our data is that while the consumer panel is representative with respect to observable characteristics such as age, region and income, there may be differences between the panel and the population in unobservable characteristics. Further, the food purchase data analyzed includes purchases for consumption at home, and any effects on out‐of‐home consumption are thus not considered. While our data prevents such analysis, future studies may explore effects from health shocks on out‐of‐home consumption and how this correlates with at home consumption.

Our study makes significant contributions to the literature by addressing weaknesses in data quality. By leveraging official health data for the identification of health shocks, including detailed diagnostic information, we provide valuable insights. However, there are limitations to our data that could potentially bias our findings. For instance, the data on food purchases rely on a consumer panel where participants are required to scan all their food purchases daily. It is likely that some items go unreported, particularly those that are less healthy and purchased spontaneously. If the likelihood of failing to report unhealthy items changes in response to health shocks, it could introduce bias into our results.

## Ethics Statement

Approval from the ethical committee on the use of data on diagnosis and the use of prescription medication has been obtained from the University of Copenhagen.

## Conflicts of Interest

The authors declare no conflicts of interest.

## Supporting information

Supporting Information S1

## Data Availability

The data that support the findings of this study are available from Restrictions apply to the availability of these data. Restrictions apply to the availability of these data, which were used under license for this study. Data are available from the author(s) with the permission of Restrictions apply to the availability of these data.

## Endnotes

^1^
The survey consists of energy intake for females and males in the age groups: 0–5, 6–9, 10–14, 15–17, 18–24, 25–34, 35–44, 45–54, 55, 64, 65+. The standard individual is constructed based on recommended nutrient requirements. The standard person chosen here is a woman at age 30—60 who has an energy requirement at 9900 KJ/day. that is a household consisting of a female and a male both aged 30—60 will have a family energy requirement of 11,000 KJ+ 9900Kj = 20,900. This household hence consists of 2.1 standard persons.

^2^
For a more detailed description of the identification of T2D see Edenbrandt et al. (2022).
