# Supplementary material for: Health Shock Effects on Diet: More Severe Shock—Stronger Response?
Source: Health Econ. 2025 Jan 22;34(5):869–79. doi: 10.1002/hec.4940 (PMC11961341; doi:10.1002/hec.4940)
Supplement: Supplementary file 1 — Supporting Information S1 [file HEC-34-869-s001.pdf]

Section S1. Healthy Eating Index and standard persons

The HEI measure is constructed similar to the Healthy Eating Index (HEI) presented in Gil et al. (2015), but aligned to the recommendations provided by the Danish Ministry of Family and Consumer Affairs. The HEI therefore evaluates the adherence to the Danish official dietary guidelines. The HEI gives a measure of the dietary healthiness of the household covering eight aspects of the diet. The construction of the HEI follows the same principles as presented in supplementary materials in Edenbrandt *et al*., (2022).

The HEI index is composed by scores, quantifying how well each household follows each of the official dietary recommendations concerning consumption of fruit and vegetables, meat, fish, dietary fibres, saturated fat, total fat, salt and added sugar as presented in Table S1.1.

Each of the households obtain a score between 0 and 1 according to how well the adhere to each of the eight recommendations. 0 is equivalent to the worst possible consumption. 1 is equivalent to that the household fully adhere to the dietary recommendation. We calculate the scores as follows:

$$Score_{int} = \left(\frac{s_{int} - s_{0,i}}{s_{recommended,ni} - s_{0,i}} \right)$$

Where s_{int} is the n'th households' consumption of a given food or nutrient *i* at the time *t*, $s_{recommended,ni}$ is the household energy requirement adjusted recommended consumption and $s_{0,i}$ is the consumption level that will yield a score of zero. Defining the consumption level for s_0 is based on the worst level of consumption observed in data, where we use the 99th percentile of consumption to define the worst level. The linearity indirectly assumed in the measure is a weakness. In reality, the marginal value for dietary quality for a piece of fruit might be greater if the initial consumption is zero compared to if the initial consumption is three pieces. The calculated scores do not encounter such differences.

Table S1.1. Operationalization of the official 2013 Danish dietary guidelines for calculation of the HEI.

Food or nutrient category	Official recommendation	Calculated recommended pct. of daily calorie intake
Fruit and vegetables	Min. 600 g per day, hereof at least 300g vegetables (300g-500g for children between 4-10years)	Min. 11.5 pct.[1)]
Fish	Min. 350 g per week	Min. 3.5 pct.[2)]
Wholegrain	Min. 75 g per day (Min 25-35 g dietary fiber) per day	Min. 2.4 pct. [3)]
Meat from beef, veal, lamb or pork	Max 500 g per week	Max. 7 pct.[4)]
Fat	Max. 25-40 pct. of the daily calorie intake	Max. 25-40 pct.
Saturated fats	Max. 10 pct. of the daily calorie intake.	Max. 10 pct.
Added sugar	Max. 10 pct. of the daily calorie intake.	Max. 10 pct.
Salt	Max 6-7 g daily	This cannot be converted into energy shares

Source: Source: The Danish Veterinary and Food Administration: www.altomkost.dk , accessed November 2020, the table is adapted from Edenbrandt et al (2022)

[1)] We calculate the recommended share of daily calorie intake as the average energy density for fruit and vegetables in the purchase data (1.96 kJ/g) multiplied by the recommended intake per day and divided by the average recommended total calorie intake in kJ per day per person (7970kJ for kids 2-17 years and 9995 kJ

for adults). In the specific HEI calculations, we use household-specific energy densities according to type of fruit and vegetable consumed in the household together with approximate energy requirements based on household composition.

2) We calculate the recommended share of daily calorie intake as the average energy density for fish in the purchase data (6.80 kJ/g) multiplied by the recommended 300g per week (≈43g. per day) and divided by the average recommended total calorie intake in kJ per day per person (7970kJ for kids 2-17 years and 9995 kJ for adults). In the specific HEI calculations, we use household-specific energy densities according to type of fish consumed in the household together with approximate energy requirements based on household composition.

3) We calculate the recommended share of daily calorie intake as the average caloric density of fibers in the purchase data (8 kJ/g) multiplied by the recommended 30g a day and divided by the average recommended total calorie intake in kJ per day per person (7970kJ for kids 2-17 years and 9995 kJ for adults).

4) We calculate the recommended share of daily calorie intake as the average energy density for meat in the purchase data (9.65 kJ/g) multiplied by the recommended 500g per week (≈71g. per day) and divided by the average recommended total calorie intake in kJ per day per person (7970kJ for kids 2-17 years and 9995 kJ for adults 18-64). In the specific HEI calculations, we use household-specific energy densities according to type of meat consumed in the household together with approximate energy requirements based on household composition.

When we have calculated the scores for each of the food and nutrients mentioned in table S1.1, they are weighted together yielding a HEI value for each household *n* at time *t* that describes how well the household adhere to the dietary recommendations. When we pool the scores into one single measure, we do not use any weights, which implies that all dietary recommendations are considered equally important for a healthy diet. This might not be completely appropriate since some recommendations might have a greater impact on dietary quality and health, especially in relation to life-style related illnesses, but identifying and justifying any differentiating in scores by ranking the dietary recommendations is not within the scope of this analysis. Furthermore, another complication of a potential weighting would be that the effects on health might be heterogeneous across individuals. We use an Euclidean distance measure to weight the individuals scores together. There are eight components in the HEI measure, denoted by *i, n* is household identification number and *t* is time:

$$HEI_{nt} = \sqrt{\sum_{1=i}^{8} (score_{int})^2}$$

The original HEI runs from 0 to $\sqrt{8}$, but we rescale the index to run from 0 to 100, for ease of interpretation.

Edenbrandt, A. K., Ewers, B., Storgaard, H., and Smed, S. (2022). Dietary changes based on food purchase patterns following a type 2 diabetes diagnosis. *Public Health Nutrition 25*: 2782–2793.

Gil, Á., de Victoria, E. M., and Olza, J. (2015). Indicators for the evaluation of diet quality. *Nutricion Hospitalaria 31*: 128–144.

Section S2. Identification of treatments

Table S2.1 Complications as registered at hospitals, and the medication prescriptions used for identification of HCP, HC and CVD

	ICD-10 codes / ATC-codes / Procedural codes
Cardiovascular disease:	
Ischemic heart disease	ICD-10: I20x - I25x.
Ischemic stroke/TCI	ICD-10: I63x, I64x, G458, G459.
Peripheral artery disease	ICD-10: I70x, I73x.
CV risk factor:	
Arterial hypertension	ICD-10:<br>I10x (I11x til I15x are complications or hypertension with known other cause, for example kidney disease).<br>And/or:<br>ATC-codes:<br>≥2 of the following medications:<br>Alfa-adrenergic blockers: C02A, C02B, C02C.<br>Non-loop diuretics: C02DA, C02L, C03A, C03B, C03D, C03E C03X, C07C, C07D, C08G, C09BA, C09DA, C09XA52.<br>Vasodilators: C02DB, C02DD, C02DG, C04x, C05x.<br>Beta-blockers: C07x.<br>Calcium channel blockers: C07F, C08, C09BB, C09DB.<br>RAS-inhibitors: C09x.
Hyperlipidemia	ICD-10: E78x.<br>ATC-codes: C10x.

Note: ”x” implies that all codes starting with code are included.

Details on the complications codes are available at https://www.dst.dk/da/TilSalg/Forskningsservice/Dokumentation/hoejkvalitetsvariable/sygehusbenyttelse---indlaeggelser-og-ambulante-behandlinger

Details on medications are available at https://www.medicinpriser.dk/Default.aspx?id=65&letter=C

Section S3. Sensitivity analysis

Table S3.1. Descriptive statistics for Control sample and Treated sample

	**Control**	**Treated**	**Test for differences (p-value)**
Personal characteristics			
# Individuals	9,672	1,151	
Female (%)	51.96	55.78	0.014
Age (mean)	38.51	64.36	<0.001
Household size (mean)	2.71	1.89	<0.001
Single household (%)	15.85	30.15	<0.001
Income in DKK (mean)	255,881	252,415	0.554
Type 2 diabetes (%)	1.26	18.68	<0.001
Food purchase patterns			
# Individuals	9,672	1,151	
HEI	76.25	77.55	<0.001
Saturated fat (E%)	14.77	15.83	<0.001
Added sugar (E%)	5.57	4.22	<0.001
Fibre (E%)	2.17	2.22	0.247
Fruit and Vegetables (E%)	8.43	6.70	<0.001
Fish (E%)	0.96	1.58	<0.001
Meat (E%)	10.33	12.47	<0.001
Sugar-sweetened beverage (E%)	2.65	1.80	<0.001

Note: Treated includes individuals that are treated or prescribed medicine for CVD, HA or HC (group b, c or d in Table 1). For age and income there are missing values in the control group, resulting in that average age is calculated using 8,449 observations and average income is calculated using 9,659 observations. For the food purchase patterns, the treated group only includes observations prior to the treatment. The calculation of the E%'s is explained in section S1.

Table S3.2. Descriptive statistics for control and treated samples (single households only)

	**Control**	**SHS-group**	**MHS-group**	**MHS+SHS-group**	**Test for difference (p-value)**
Personal characteristics					
# Individuals	1,533	33	280	34	
Female (%)	69.86	81.82	83.57	64.71	<0.001
Age (mean)	40.63	65.91	66.92	69.65	<0.001
Income in DKK (mean)	284,942	252,191	262,510	247,771	0.024
Type 2 diabetes (%)	1.63	9.09	22.14	23.53	<0.001
Food purchase patterns					
HEI	78.03	79.10	79.27	78.63	0.100
Saturated fat (E%)	14.38	15.96	15.89	16.13	0.001
Added sugar (E%)	6.01	4.79	4.87	3.50	0.003
Fibre (E%)	2.23	2.35	2.30	2.18	0.834
Fruit and Vegetables (E%)	9.27	7.91	8.22	4.85	0.192
Fish (E%)	1.10	1.44	1.61	1.66	0.179
Meat (E%)	8.80	11.62	11.53	11.20	<0.001
Sugar-sweetened beverage (E%)	3.03	2.04	1.71	2.16	0.019

Note: There are missing values in the control group for age, resulting in that average age is calculated using only 1,532 observations. For the food purchase patterns, the treated group only includes observations prior to the treatment. The calculation of the E%'s is explained in Section S1.

Table S3.3. Descriptive statistics for control and treated samples (individuals above 55 years only)

	**Control**	**SHS-group**	**MHS-group**	**MHS+SHS-group**	**Test for difference (p-value)**
Personal characteristics					
# Individuals	1,198	105	789	99	
Female (%)	53.59	41.90	60.46	43.43	<0.001
Age (mean)	61.69	66.17	67.82	70.31	<0.001
Household size (mean)	1.90	1.80	1.77	1.75	<0.001
Single household (%)	25.96	29.52	31.05	31.31	0.082
Income in DKK (mean)	315,132	242,701	243,748	221,471	<0.001
Type 2 diabetes (%)	0.58	11.43	18.50	21.21	<0.001
Food purchase patterns					
HEI	78.19	78.18	78.14	78.81	0.844
Saturated fat (E%)	15.27	15.71	15.89	15.59	0.025
Added sugar (E%)	4.82	4.48	4.18	3.89	0.001
Fibre (E%)	2.27	2.36	2.24	2.30	0.665
Fruit and Vegetables (E%)	8.48	7.35	6.92	6.14	0.003
Fish (E%)	1.62	1.33	1.76	1.73	0.647
Meat (E%)	10.67	11.83	12.47	10.99	<0.001
Sugar-sweetened beverage (E%)	1.48	1.53	1.61	1.78	0.845

Note: There are missing values for income, resulting that only 1,197 observations are used to calculate average income for the control group and only 98 observations to calculate the average for the MHS+SHS group. For the food purchase patterns, the treated group only includes observations prior to the treatment. The number of observations that is used to calculate the averages across food purchase patterns are 96, 698 and 90 respectively for the treatment groups. The calculation of the E%'s is explained in Section S1.

Table S3.4 Descriptive statistics for length (measured in months) of participation in panel

	# Individuals	Mean	Std. Dev	Minimum	Maximum
Full sample					
Control group	9672	21.8	36.1	1	144
SHS group	119	104.2	44.9	2	144
MHS group	925	95.4	47.9	2	144
MHS+SHS group	107	106.4	40.5	9	144
Above 55 years only					
Control group	1035	49.2	53.3	1	144
SHS group	95	108.7	41.8	11	144
MHS group	702	97.2	47	3	144
MHS+SHS group	93	106.1	38.8	9	144

Table 3.5 Naïve specification for effect of health shocks on dietary patterns

	HEI	F&V	Fish	Red meat	Sat fat	Fibre
Mild health shock effects						
MHS	0.54***	0.11	0.18**	-0.30	-0.16	0.01
	(2.34)	(0.64)	(1.69)	(1.11)	(1.16)	(0.54)
MHS_{-3}	-0.33*	-0.01	-0.33***	0.02	0.03	0.00
	(1.51)	(0.04)	(3.63)	(0.07)	(0.23)	(0.13)
MHS_{-6}	-0.15	-0.36***	0.08*	-0.09	0.15*	-0.03*
	(0.83)	(2.47)	(1.35)	(0.43)	(1.43)	(1.47)
Strong health shock effects						
SHS	0.15	0.61	0.12	0.45	-0.12	0.07*
	(0.37)	(2.16)	(0.89)	(0.99)	(0.48)	(1.46)
SHS_{-3}	0.83**	-0.39	0.07	-0.89**	-0.23	-0.01
	(2.04)	(1.21)	(0.40)	(1.97)	(0.96)	(0.18)
SHS_{-6}	-0.82***	0.41*	-0.08	0.40*	0.28*	-0.08**
	(2.57)	(1.34)	(0.64)	(1.32)	(1.42)	(1.92)
Control variables						
T2D	1.82***	1.24**	0.34*	-1.28*	-0.70**	0.12**
	(3.20)	(1.92)	(1.59)	(1.64)	(2.11)	(1.73)
$T2D_{-3}$	0.02	-0.27	-0.15	-0.40	0.05	-0.01
	(0.04)	(0.88)	(0.75)	(0.62)	(0.18)	(0.21)
$T2D_{-6}$	-1.46***	-0.36	-0.20	0.83**	0.56**	-0.09**
	(3.75)	(0.73)	(1.22)	(1.91)	(2.32)	(2.12)
Lead variables						
MHS_{+3}	0.22	0.23*	0.04	0.09	-0.13	0.03*
	(1.12)	(1.38)	(0.45)	(0.44)	(1.09)	(1.64)
SHS_{+3}	0.43*	0.36	0.15	-0.10	-0.08	0.01
	(1.41)	(1.20)	(1.21)	(0.25)	(0.43)	(0.19)
$T2D_{+3}$	-0.65*	0.20	-0.22*	0.52	0.06	0.00
	(1.45)	(0.32)	(1.37)	(0.91)	(0.20)	(0.04)
Intercept	79.66***	7.48***	1.39***	11.64***	14.62***	2.40***
	(686.85)	(77.94)	(34.13)	(99.32)	(214.66)	(200.44)
Within R^2	0.02	0.01	0.01	0.01	0.02	0.02
F-statistic	83.93	54.63	23.91	17.81	89.65	104.74
Test MHS[a] (p-value)	0.774	0.139	0.471	0.131	0.868	0.595
Test SHS[b] (p-value)	0.659	0.061	0.492	0.934	0.727	0.737

Note: |t-values| in parenthesis P-values for one-sides tests are indicated by *p<0.10, ****p< 0.05, ***** p< 0.01. Year and month variables included in all models. N=262,761. Individuals=4,780. [a]$MHS+MHS_{-3}+MHS_{-6}=0$. [b]$SHS+SHS_{-3}+SHS_{-6}=0$

Table S3.6 Descriptive statistics for change in HEI following treatment and pre-treatment HEI

	Dietary change following treatment ($\tilde{y}_i$)[a]		Pre-treatment ($\bar{y}_{i,pre-treatment}$)[b]	
	MHS	SHS	MHS	SHS
# Individuals	1010	219	1030	226
Mean	-0.07	0.88	77.55	77.38
Std, dev,	5.39	4.83	7.24	6.35
Percentiles				
1%	-12.82	-9.56	58.02	62.78
5%	-8.17	-6.34	65.28	66.44
10%	-6.43	-4.74	68.26	69.26
25%	-3.27	-2.34	72.74	72.78
50%	-0.10	0.84	77.87	77.73
75%	3.19	3.74	82.58	81.84
90%	6.17	7.29	86.37	85.57
95%	8.19	9.73	88.84	87.90
99%	14.87	14.77	92.73	91.23

[a] $\tilde{y}_i = \bar{y}_{i,post-treatment} - \bar{y}_{i,pre-treatment}$, such that $\tilde{y}_i$ is the difference between the average dietary outcome measured using the HEI in the three months following treatment compared to the average in the three months preceding it. [b] $\bar{y}_{i,pre-treatment}$ is the average HEI in the three months prior to the treatment.

Table. S.3.7 Regression with the change in HEI following health shock treatment as the dependent variable regressed on the pre-treatment HEI

	MHS	SHS
Pre-treatment HEI	-0.31***	-0.24***
	(12.46)	(-4.62)
Intercept	24.00***	19.16***
	(12.21)	(4.74)
Observations	1010	219
R^2	0.17	0.09
F-statistic	155.25	21.34

Note: $\tilde{y}_i = \beta_0 + \beta_1 \bar{y}_{i,pre-treatment_i} + \varepsilon_i$ (equation 2 in manuscript). t-values in brackets. P-values for two-sides tests are indicated by *p<0.10, **p< 0.05, *** p< 0.01.

Table S3.8 Quantile regression with the change in HEI following health shock treatment regressed on the pre-treatment HEI level.

	MHS		SHS	
	Coefficient	|t-value|	Coefficient	|t-value|
Quantile 10				
Pre-treatment	-0.26***	(6.33)	-0.06	(0.61)
Intercept	14.27***	(4.48)	-0.28	(-0.04)
Quantile 20				
Pre-treatment	-0.25***	(9.26)	-0.13**	(2.20)
Intercept	15.70***	(7.40)	7.31	(1.55)
Quantile 30				
Pre-treatment	-0.26***	(8.57)	-0.13**	(2.28)
Intercept	18.04***	(7.70)	8.38*	(1.85)
Quantile 40				
Pre-treatment	-0.25***	(9.26)	-0.22***	(3.18)
Intercept	18.09***	(8.73)	16.46***	(3.04)
Quantile 50				
Pre-treatment	-0.26***	(9.32)	-0.22***	(2.98)
Intercept	20.53***	(9.31)	17.87***	(3.12)
Quantile 60				
Pre-treatment	-0.28***	(11.68)	-0.26***	(2.94)
Intercept	22.46***	(12.30)	22.18***	(3.19)
Quantile 70				
Pre-treatment	-0.27***	(7.75)	-0.27***	(5.68)
Intercept	23.59***	(8.56)	24.65***	(6.44)
Quantile 80				
Pre-treatment	-0.30***	(8.74)	-0.28***	(7.04)
Intercept	27.23***	(9.69)	25.78***	(8.16)
Quantile 90				
Pre-treatment	-0.36***	(10.15)	-0.37***	(5.66)
Intercept	34.02***	(11.66)	34.77***	(6.37)
Individuals	1010		219	

Note: t-values in brackets. p-values for two-sides tests are indicated by *p<0.10, **p< 0.05, *** p< 0.01.

Figure S3.1 Quantile regression with change in HEI following MHS regressed on pre-treatment level of HEI

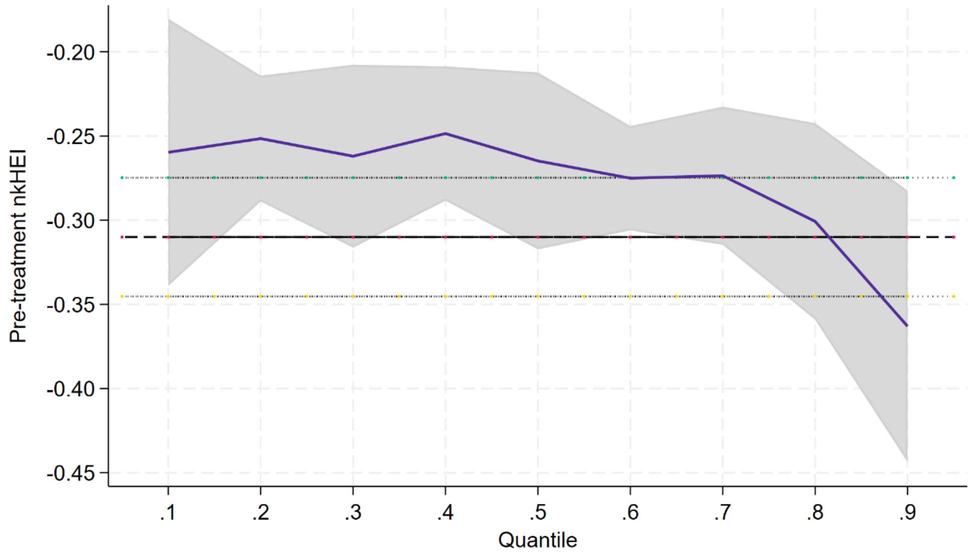

Note: Horizontal black line is the OLS-regression (Table S3.6) with dotted lines for 95% confidence intervals. The purple line is the quantile regression (Table S3.7), where the shaded areas is a 95% confidence interval

Figure S3.2 Quantile regression with change in HEI following SHS regressed on pre-treatment level of HEI

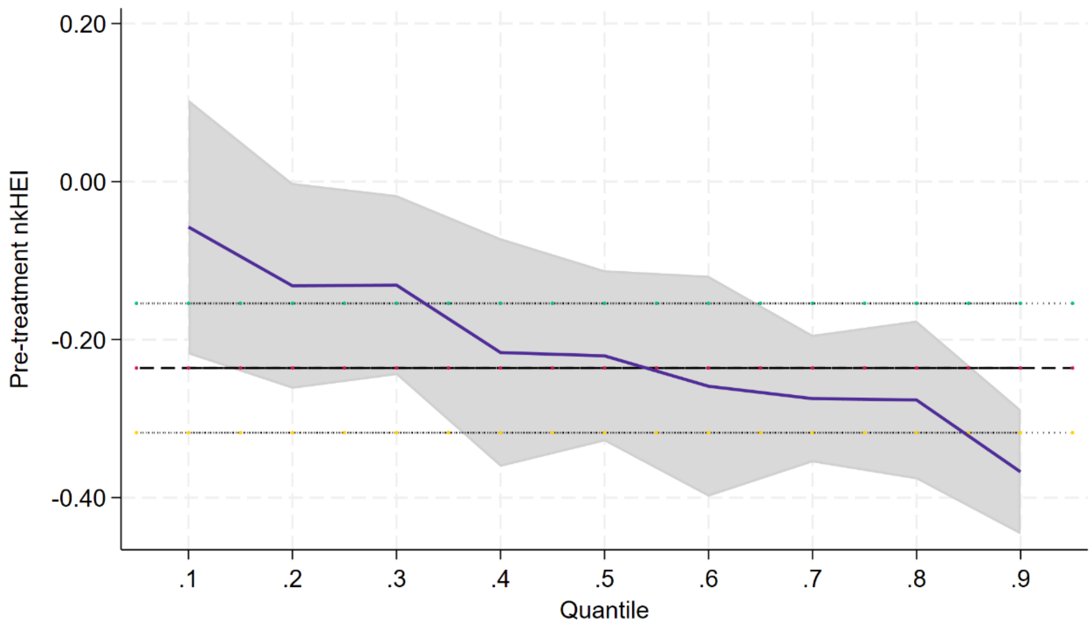

Note: Horizontal black line is the OLS-regression (Table S3.6) with dotted lines for 95% confidence intervals. The purple line is the quantile regression (Table S3.7), where the shaded areas is a 95% confidence interval

Table S3.9. Effect of health shocks on dietary patterns (single households only)

	HEI	F&V	Fish	Red meat	Sat fat	Fiber
Mild health shock effects						
MHS	0.84**	-0.01	-0.11	-0.35	-0.42*	0.01
	(1.96)	(0.02)	(0.52)	(0.82)	(1.44)	(0.23)
MHS_{-3}	-0.70**	-0.40*	-0.32**	0.41	0.32	-0.01
	(1.68)	(1.28)	(2.10)	(0.92)	(1.15)	(0.28)
MHS_{-6}	-0.20	-0.17	0.10	0.13	0.32*	-0.03
	(0.61)	(0.61)	(0.98)	(0.36)	(1.32)	(0.70)
Strong health shock effects						
SHS	2.02**	1.00	0.51	-0.51	-1.56**	0.23*
	(2.07)	(1.16)	(1.07)	(0.60)	(2.13)	(1.60)
SHS_{-3}	1.33*	-0.77	0.02	-0.62	0.27	-0.03
	(1.31)	(0.64)	(0.03)	(0.79)	(0.45)	(0.30)
SHS_{--6}	-2.12***	-0.28	-0.27	0.75	0.53	-0.28***
	(2.42)	(0.26)	(0.66)	(1.04)	(1.04)	(2.46)
SHS*MHS	-3.48***	-0.54	-0.48	1.87**	2.18**	-0.46***
	(2.36)	(0.49)	(0.65)	(1.83)	(2.14)	(2.56)
SHS_{-3}*MHS	-0.49	-0.11	-0.55	1.05	0.06	0.23*
	(0.33)	(0.09)	(0.69)	(0.80)	(0.08)	(1.58)
SHS_{-6}*MHS	2.29**	0.81	0.38	-1.65*	-1.19*	0.23*
	(2.02)	(0.63)	(0.78)	(1.44)	(1.64)	(1.42)
Control variables						
T2D	2.69***	0.48	1.03***	-1.56*	-0.58	0.13
	(2.37)	(0.56)	(2.37)	(1.50)	(0.85)	(1.09)
$T2D_{-3}$	-0.46	-0.04	-0.66*	-0.42	-0.20	0.03
	(0.49)	(0.06)	(1.62)	(0.35)	(0.31)	(0.33)
$T2D_{-6}$	-1.88	-0.64	-0.41*	1.77**	0.72*	-0.15**
	(2.57)	(0.88)	(1.37)	(2.02)	(1.44)	(2.03)
Lead variables						
MHS_{+3}	0.01	0.39	0.25*	-0.01	0.02	0.02
	(0.04)	(0.90)	(1.38)	(0.04)	(0.10)	(0.51)
SHS_{+3}	0.57	1.20**	0.62**	-0.33	-0.39	0.13
	(1.10)	(2.01)	(1.91)	(0.51)	(0.85)	(1.57) *
$T2D_{+3}$	-1.28*	0.33	-0.39	1.22	0.60	-0.06
	(1.44)	(0.28)	(1.06)	(1.23)	(0.96)	(0.56)
Intercept	81.45***	8.80***	1.75***	10.69***	14.76***	2.49***
	(334.61)	(34.34)	(15.59)	(42.59)	(90.75)	(79.69)
Within R^2	0.02	0.01	0.01	0.01	0.02	0.01
F-statistic	15.03	10.48	5.77	5.94	18.45	16.34
Test MHS[a] (p-values)	0.870	0.209	0.074	0.656	0.412	0.536
Test SHS[b] (p-values)	0.141	0.957	0.705	0.657	0.227	0.445

Note: |t-values| in parenthesis. p-values for one-sides tests are indicated by *p<0.10, **p< 0.05, *** p< 0.01. Year and month variables included in all models. N=59,203. Individuals=1,034. [a]$MHS+MHS_{-3}+MHS_{-6}=0$. [b]$SHS+SHS_{-3}+SHS_{-6}=0$

Table S3.10. Effect of health shocks on dietary patterns (constant household size sample)

	HEI	F&V	Fish	Red meat	Sat fat	Fiber
Mild health shock effects						
MHS	0.68***	0.08	0.25**	-0.47*	-0.28**	0.01
	(2.65)	(0.40)	(1.95)	(1.53)	(1.85)	(0.33)
MHS_{-3}	-0.29	-0.22*	-0.33***	0.14	0.03	0.00
	(1.20)	(1.43)	(2.93)	(0.48)	(0.19)	(0.10)
MHS_{-6}	-0.36**	-0.21*	0.08	0.02	0.28***	-0.03*
	(1.82)	(1.55)	(1.15)	(0.07)	(2.50)	(1.43)
Strong health shock effects						
SHS	0.44	0.90*	-0.04	-0.75	-0.52	0.17**
	(0.69)	(1.64)	(0.17)	(1.21)	(1.22)	(2.18)
SHS_{-3}	0.96*	-0.35	0.00	0.15	-0.21	-0.06
	(1.52)	(0.54)	(0.01)	(0.29)	(0.57)	(0.79)
SHS_{-6}	-0.67*	0.97*	-0.09	0.15	0.10	-0.09*
	(1.40)	(1.47)	(0.40)	(0.32)	(0.34)	(1.45)
SHS*MHS	-0.56	-0.58	0.11	2.62***	0.46	-0.14*
	(0.65)	(0.78)	(0.35)	(3.08)	(0.88)	(1.34)
SHS_{-3}*MHS	-0.27	0.36	0.04	-2.41***	0.35	0.06
	(0.30)	(0.47)	(0.11)	(2.44)	(0.62)	(0.56)
SHS_{-6}*MHS	-0.14	-1.09*	-0.04	0.53	0.04	0.05
	(0.20)	(1.40)	(0.13)	(0.80)	(0.10)	(0.56)
Control variables						
T2D	1.73***	1.96**	0.46*	-1.83**	-0.38	0.09
	(2.41)	(2.15)	(1.60)	(1.83)	(0.93)	(1.05)
$T2D_{-3}$	-0.43	-0.76**	-0.23	0.44	-0.03	-0.01
	(0.81)	(1.91)	(0.81)	(0.61)	(0.09)	(0.25)
$T2D_{-6}$	-1.16***	-0.36	-0.23	0.70*	0.46*	-0.07*
	(2.53)	(0.54)	(1.01)	(1.44)	(1.62)	(1.31)
Lead variables						
MHS_{+3}	0.30*	0.14	0.00	0.00	-0.11	0.04**
	(1.41)	(0.75)	(0.01)	(0.01)	(0.85)	(1.77)
SHS_{+3}	0.59**	0.35	0.15	-0.10	-0.13	0.01
	(1.81)	(1.02)	(1.10)	(0.21)	(0.61)	(0.26)
$T2D_{+3}$	-0.40	0.44	-0.24	0.84	0.02	0.05
	(0.72)	(0.53)	(1.12)	(1.13)	(0.05)	(0.72)
Intercept	80.41***	7.77***	1.51***	11.90***	14.77***	2.47***
	(567.90)	(61.26)	(26.89)	(82.39)	(175.21)	(162.22)
Within R^2	0.02	0.01	0.01	0.01	0.02	0.02
F-statistic	54.39	35.73	17.25	14.08	61.16	62.58
Test MHS[a] (p-values)	0.904	0.076	0.985	0.258	0.840	0.344
Test SHS[b] (p-values)	0.117	0.037	0.477	0.344	0.053	0.740

Note: |t-values| in parenthesis. p-values for one-sides tests are indicated by *p<0.10, **p< 0.05, *** p< 0.01. Year and month variables included in all models. N=171,633. Individuals=3327. [a]$MHS+MHS_{-3}+MHS_{-6}=0$. [b]$SHS+SHS_{-3}+SHS_{-6}=0$

Table S3.11. Effect of health shocks on dietary patterns (individuals above 55 years only)

	**HEI**	**F&V**	**Fish**	**Red meat**	**Sat fat**	**Fiber**
Mild health shock effects						
MHS	0.42*	0.01	0.18*	-0.21	-0.17	0.00
	(1.63)	(0.04)	(1.33)	(0.76)	(1.14)	(0.09)
MHS_{-3}	-0.22	-0.09	-0.39***	-0.16	0.00	0.02
	(0.91)	(0.55)	(3.58)	(0.60)	(0.01)	(0.68)
MHS_{-6}	-0.27*	-0.40***	0.13**	0.11	0.22**	-0.04
	(1.33)	(2.58)	(1.80)	(0.53)	(2.00)	(1.73)
Strong health shock effects						
SHS	0.42	0.98**	-0.08	-0.33	-0.59*	0.15**
	(0.78)	(1.89)	(0.40)	(0.53)	(1.51)	(1.97)
SHS_{-3}	0.74*	-0.68	0.29	-0.20	-0.04	-0.07
	(1.33)	(1.09)	(0.98)	(0.36)	(0.10)	(1.16)
SHS_{-6}	-1.01**	0.60	-0.16	0.25	0.24	-0.14**
	(2.07)	(1.01)	(0.72)	(0.56)	(0.82)	(2.26)
SHS*MHS	-0.65	-1.01*	0.18	1.85***	0.54	-0.16**
	(0.91)	(1.51)	(0.62)	(2.38)	(1.17)	(1.75)
SHS_{-3}*MHS	-0.09	0.71	-0.39	-1.36*	0.23	0.10
	(0.11)	(1.00)	(1.08)	(1.49)	(0.43)	(0.96)
SHS_{-6}*MHS	0.26	-0.74	0.16	0.07	-0.07	0.08
	(0.39)	(1.07)	(0.60)	(0.12)	(0.17)	(0.94)
Control variables						
T2D	1.76***	1.32*	0.49**	-0.57	-1.08***	0.13*
	(2.52)	(1.40)	(1.71)	(0.58)	(2.78)	(1.60)
$T2D_{-3}$	-0.13	-0.31	-0.17	-0.55	0.26	-0.04
	(0.23)	(0.84)	(0.70)	(0.69)	(0.72)	(0.70)
$T2D_{-6}$	-1.33***	-0.32	-0.33**	0.57	0.64**	-0.05
	(2.91)	(0.48)	(1.69)	(1.06)	(2.18)	(0.97)
Lead variables						
MHS_{+3}	0.22	0.18	0.04	0.02	-0.09	0.03*
	(0.98)	(0.93)	(0.39)	(0.10)	(0.73)	(1.44)
SHS_{+3}	0.47*	0.22	0.27**	-0.20	-0.03	0.02
	(1.52)	(0.64)	(2.02)	(0.50)	(0.13)	(0.42)
$T2D_{+3}$	-0.88*	0.56	-0.27	0.71	0.47*	-0.01
	(1.61)	(0.67)	(1.26)	(0.99)	(1.54)	(0.14)
Intercept	81.15***	7.99***	1.75***	12.27***	14.63***	2.54***
	(469.84)	(53.59)	(24.12)	(72.64)	(143.01)	(134.29)
Within R^2	0.03	0.02	0.01	0.01	0.03	0.03
F-statistic	46.35	35.82	16.02	13.25	55.03	57.62
Test MHS[a] (p-values)	0.775	0.018	0.465	0.366	0.688	0.382
Test SHS[b] (p-values)	0.719	0.178	0.856	0.567	0.199	0.285

Note: |t-values| in parenthesis. p-values for one-sides tests are indicated by *p<0.10, **p< 0.05, *** p< 0.01. Year and month variables included in all models.. N=125,295. Individuals=1755. [a]$MHS+MHS_{-3}+MHS_{-6}=0$. [b]$SHS+SHS_{-3}+SHS_{-6}=0$

Table S3.12. Effect of health shocks on dietary patterns (observations up to five months post treatment only)

	HEI	F&V	Fish	Red meat	Sat fat	Fiber
Mild health shock effects						
MHS	0.49**	0.12	0.17*	-0.31	-0.16	0.01
	(2.15)	(0.69)	(1.59)	(1.15)	(1.17)	(0.37)
MHS_{-3}	-0.36*	-0.02	-0.31***	0.06	-0.02	0.00
	(1.60)	(0.13)	(3.33)	(0.21)	(0.12)	(0.15)
MHS_{-6}	-0.48	0.27	0.23*	0.50	0.45*	-0.03
	(0.80)	(0.93)	(1.61)	(1.09)	(1.39)	(0.54)
Strong health shock effects						
SHS	0.80*	1.21***	0.11	-0.50	-0.47*	0.16***
	(1.50)	(2.84)	(0.64)	(0.87)	(1.37)	(2.55)
SHS_{-3}	0.94**	-0.75*	0.13	-0.37	-0.49*	-0.04
	(1.73)	(1.37)	(0.46)	(0.63)	(1.54)	(0.61)
SHS_{-6}						
SHS*MHS	-1.23**	-1.12**	0.02	1.71**	0.55*	-0.20***
	(1.71)	(2.13)	(0.07)	(2.53)	(1.29)	(2.40)
SHS_{-3}*MHS	-0.13	0.77	-0.11	-0.77	0.51	0.08
	(0.16)	(1.20)	(0.32)	(0.91)	(1.06)	(0.85)
SHS_{-6}*MHS						
Control variables						
T2D	2.06***	1.61***	0.42*	-1.23*	-0.77**	0.18**
	(2.81)	(3.49)	(1.32)	(1.32)	(1.74)	(2.07)
$T2D_{-3}$	0.67	-0.56*	-0.14	-1.19*	-0.03	0.00
	(1.10)	(1.30)	(0.47)	(1.32)	(0.08)	(0.06)
$T2D_{-6}$	-2.87***	-0.72*	-0.43**	1.61***	1.04***	-0.12*
	(4.29)	(1.48)	(1.67)	(2.55)	(3.23)	(1.45)
Lead variables						
MHS_{+3}	0.30*	0.37**	0.04	0.15	-0.17*	0.03**
	(1.55)	(2.32)	(0.45)	(0.72)	(1.47)	(1.67)
SHS_{+3}	0.35	0.17	0.16*	-0.13	0.01	0.00
	(1.20)	(0.58)	(1.35)	(0.33)	(0.08)	(0.03)
$T2D_{+3}$	-0.97*	-0.44	-0.29*	0.84*	0.27	-0.02
	(1.59)	(0.78)	(1.37)	(1.28)	(0.71)	(0.35)
Intercept	79.50***	7.47***	1.31***	11.37***	14.53***	2.37***
	(622.89)	(74.16)	(30.12)	(86.38)	(195.10)	(180.90)
Within R^2	0.02	0.01	0.01	0.00	0.02	0.02
F-statistic	65.05	41.90	17.28	10.85	66.78	83.37
Test MHS[a] (p-values)	0.595	0.236	0.547	0.618	0.412	0.670
Test SHS[b] (p-values)	0.002	0.292	0.430	0.089	0.006	0.068

Note: |t-values| in parenthesis. p-values for one-sides tests are indicated by *p<0.10, **p< 0.05, *** p< 0.01. Year and month variables included in all models. N=205,962 individuals=4779. [a]$MHS+MHS_{+3}+MHS_{+6}=0$ [b]$SHS+SHS_{+3}+SHS_{+6}=0$

Table S3.13 Effect of health shocks on HEI by length of participation

	SHS-group			MHS-group			MHS+SHS-group		
	All	Below Median	Above median	All	Below median	Above median	All	Below median	Above median
MHS effects									
MHS				0.58***	-0.06	1.00***	-0.05	1.84*	-1.60*
				(2.45)	(0.15)	(3.32)	(0.06)	(1.55)	(1.35)
MHS$_{-3}$				-0.38**	-0.39	-0.38**	0.19	-0.90	1.12
				(1.68)	(1.12)	(1.28)	(0.26)	(0.91)	(1.11)
MHS$_{-6}$				-0.10	-0.18	-0.05	-0.39	0.11	-0.82
				(0.51)	(0.65)	(0.21)	(0.73)	(0.14)	(1.10)
SHS effects									
SHS	0.51	-0.45	1.32**				-0.13	-0.63	0.43
	(0.92)	(0.58)	(1.71)				(0.22)	(0.87)	(0.50)
SHS$_{-3}$	0.94**	1.68**	0.35				0.76	0.70	0.83
	(1.75)	(2.04)	(0.50)				(1.25)	(0.70)	(1.14)
SHS$_{-6}$	-1.10***	-0.47	-1.51***				-0.51	-0.96*	-0.18
	(2.48)	(0.66)	(2.72)				(1.13)	(1.40)	(0.32)
Control variables									
T2D	1.74*	2.17**	2.31**	2.27***	2.65***	2.22***	1.68*	3.05***	1.24
	(1.55)	(1.69)	(1.86)	(3.55)	(2.88)	(2.92)	(1.51)	(2.32)	(1.07)
T2D$_{-3}$	-0.33	-0.14	-0.59	-0.04	-0.37	0.11	-0.08	-1.08	0.52
	(0.38)	(0.13)	(0.61)	(0.07)	(0.50)	(0.19)	(0.08)	(0.90)	(0.59)
T2D$_{-6}$	-2.23***	-2.84***	-2.60***	-1.69***	-1.89***	-1.82***	-1.90**	-2.41**	-2.46***
	(2.42)	(2.42)	(2.59)	(3.89)	(2.81)	(3.47)	(2.21)	(2.08)	(2.78)
Lead variables									
MHS$_{+3}$				0.18	0.78***	-0.22	0.84*	-0.63	1.98***
				(0.85)	(2.39)	(0.85)	(1.35)	(0.63)	(2.84)
SHS$_{+3}$	0.73**	0.51	0.86*				-0.01	0.73*	-0.84*
	(1.75)	(0.76)	(1.59)				(0.04)	(1.35)	(1.36)
DT2$_{+3}$	-0.30	-1.14	-0.47	-0.91**	-0.62	-1.17**	-0.80	-1.36*	-0.70
	(0.32)	(1.18)	(0.45)	(1.79)	(0.81)	(2.03)	(0.89)	(1.31)	(0.74)
Intercept	79.66***	79.58***	79.65***	79.60***	79.52***	79.64***	79.57***	79.56***	79.57***
	(539.49)	(524.50)	(534.24)	(649.33)	(561.40)	(613.74)	(545.75)	(529.61)	(535.95)
Observations	172,231	164,568	168,546	240,974	181,880	219,977	171,322	164,621	167,584
Individuals	3,789	3,731	3,730	4,557	4,097	4,132	3,778	3,727	3,723
Within R^2	0.02	0.02	0.02	0.02	0.02	0.02	0.02	0.02	0.02
F-statistic	60.10	57.24	59.10	87.27	63.27	79.17	52.27	50.15	51.72
Test MHS[a] (p-values)							0.758	0.326	0.221
Test SHS[b] (p-values)	0.474	0.354	0.797	0.645	0.074	0.041	0.800	0.195	0.113

Note: |t-values| in parenthesis. p-values for one-sides tests are indicated by *p<0.10, **p< 0.05, *** p< 0.01. Year and month variables included in all models.

For each group (SHS, MHS, MHS+SHS) the treated individuals are split in two groups by the number of months they report to the panel.
